# Merits and Pitfalls of Currently Used Diagnostic Tools in Mycetoma

**DOI:** 10.1371/journal.pntd.0002918

**Published:** 2014-07-03

**Authors:** Wendy W. J. van de Sande, Ahmed H. Fahal, Michael Goodfellow, El Sheikh Mahgoub, Oliverio Welsh, Ed E. Zijlstra

**Affiliations:** 1 ErasmusMC, Department of Medical Microbiology & Infectious Diseases, Rotterdam, The Netherlands; 2 Mycetoma Research Centre, University of Khartoum, Soba University Hospital, Sudan; 3 School of Biology, University of Newcastle, Newcastle upon Tyne, United Kingdom; 4 Dr. Jose E Gonzalez University Hospital, Universidad Autónoma de Nuevo León, Department of Dermatology, Ave Madero y Ave Gonzalitos, Colonia Mitras Centro, Monterrey, Nuevo Leon, Mexico; 5 Rotterdam Centre for Tropical Medicine, Rotterdam, The Netherlands; University of Tennessee, United States of America

## Abstract

Treatment of mycetoma depends on the causative organism and since many organisms, both actinomycetes (actinomycetoma) and fungi (eumycetoma), are capable of producing mycetoma, an accurate diagnosis is crucial. Currently, multiple diagnostic tools are used to determine the extent of infections and to identify the causative agents of mycetoma. These include various imaging, cytological, histopathological, serological, and culture techniques; phenotypic characterisation; and molecular diagnostics. In this review, we summarize these techniques and identify their merits and pitfalls in the identification of the causative agents of mycetoma and the extent of the disease. We also emphasize the fact that there is no ideal diagnostic tool available to identify the causative agents and that future research should focus on the development of new and reliable diagnostic tools.

## Introduction

**Figure 1:**
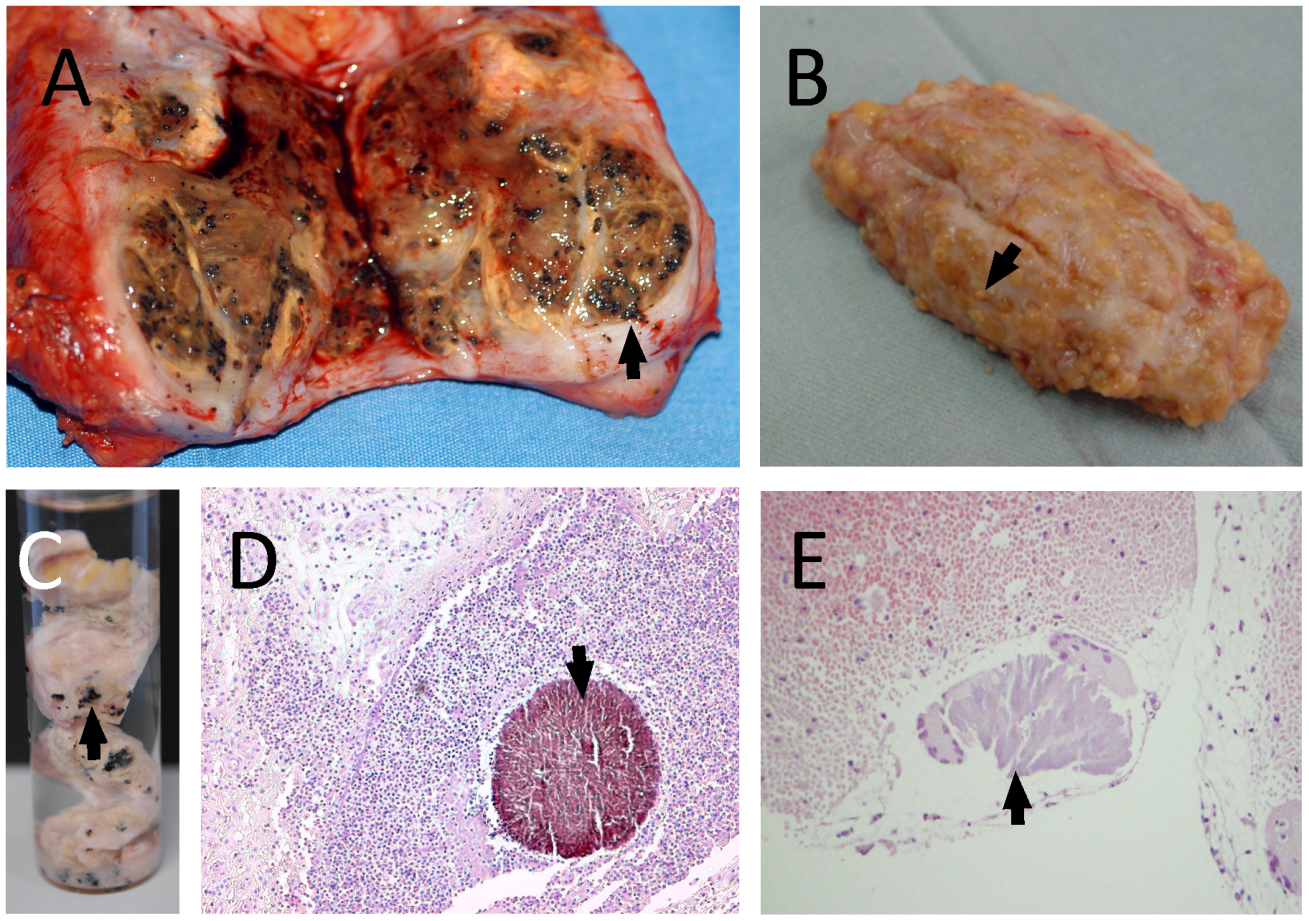
Different grains obtained from eumycetoma and actinomycetoma lesions. In this figure different grain types are shown. A: An eumycetoma surgical excision with numerous black grains, indicative for *M. mycetomatis*. B: An actinomycetoma surgical biopsy with numerous yellow grains, indicative for *S. somaliensis*. C: Grains of *Madurella mycetomatis* fixed in formalin. D: Histological slide of a *Madurella mycetomatis* grain inside subcutaneous tissue. The grain is clearly seen as a round brown structure (arrow) (×100). E: Histological slide of a *S. somaliensis* grain inside subcutaneous tissue (arrow) (×400).

Mycetoma is a common medical and health problem in many tropical and subtropical regions. It is an infection of the subcutaneous tissues that is caused by actinomycetes (actinomycetoma) or fungi (eumycetoma) and is characterized by the development of large subcutaneous masses and discharging sinuses [1]. Within the discharged material, the causative agents are located both in and outside of grains. The colours of the grains can often be indicative of the aetiological agent: fungal grains are usually black or pale, while those of actinomycetes are white, yellow, or red (Figure 1) [1].

Since both actinomycetes and fungi are implicated as causative agents, it is important to distinguish them in order to ensure that correct treatments are given. Actinomycetoma is treated with antibiotics and eumycetoma with a combination of antifungal therapy and surgery [2]. The relevance of correct species identification was emphasised in a recent published case report in which a patient presented with yellow-grain eumycetoma caused by the fungus *Pleurostomophora ochracea* [3]. In the past, yellow grains were always considered to be of bacterial origin, and antibacterial, as opposed to antifungal, treatment was started and found to be ineffective.

In addition to differentiating actinomycetoma and eumycetoma, it is important to identify causal agents to the species level to ensure correct antimicrobial therapy. Several actinomycetes and fungi have been implicated as causative agents of mycetoma [4]. In vitro, not all fungi are equally susceptible to antifungal agents; for instance, *Madurella fahalii* is not inhibited by high concentrations of ketoconazole and itraconazole, which are drugs that are used in the treatment of eumycetoma [5]. Regarding actinomycetoma, therapeutic outcome is based on a prompt and adequate response to the administered antimicrobials. Actinomycetes usually respond well to cotrimoxazole, but a combination of this antibiotic with aminoglycosides is used in advanced cases or in cases not responding to treatment. Other antibiotics can also be used. Ideally, sensitivity tests should be done before starting treatment, namely, shortly after the etiologic agent is isolated.

In this review we summarize the currently used diagnostic tools and discuss their merits and pitfalls in the diagnosis of mycetoma.

## Imaging Technology

An initial diagnosis is made after clinical assessment. Clinical examination alone does not identify the causative organism nor does it detect the spread of disease along the different tissue planes and bone. Imaging techniques, such as radiology, ultrasonography, CT scan, and magnetic resonance imaging (MRI) can be used to determine the extent of lesions.

**Figure 2:**
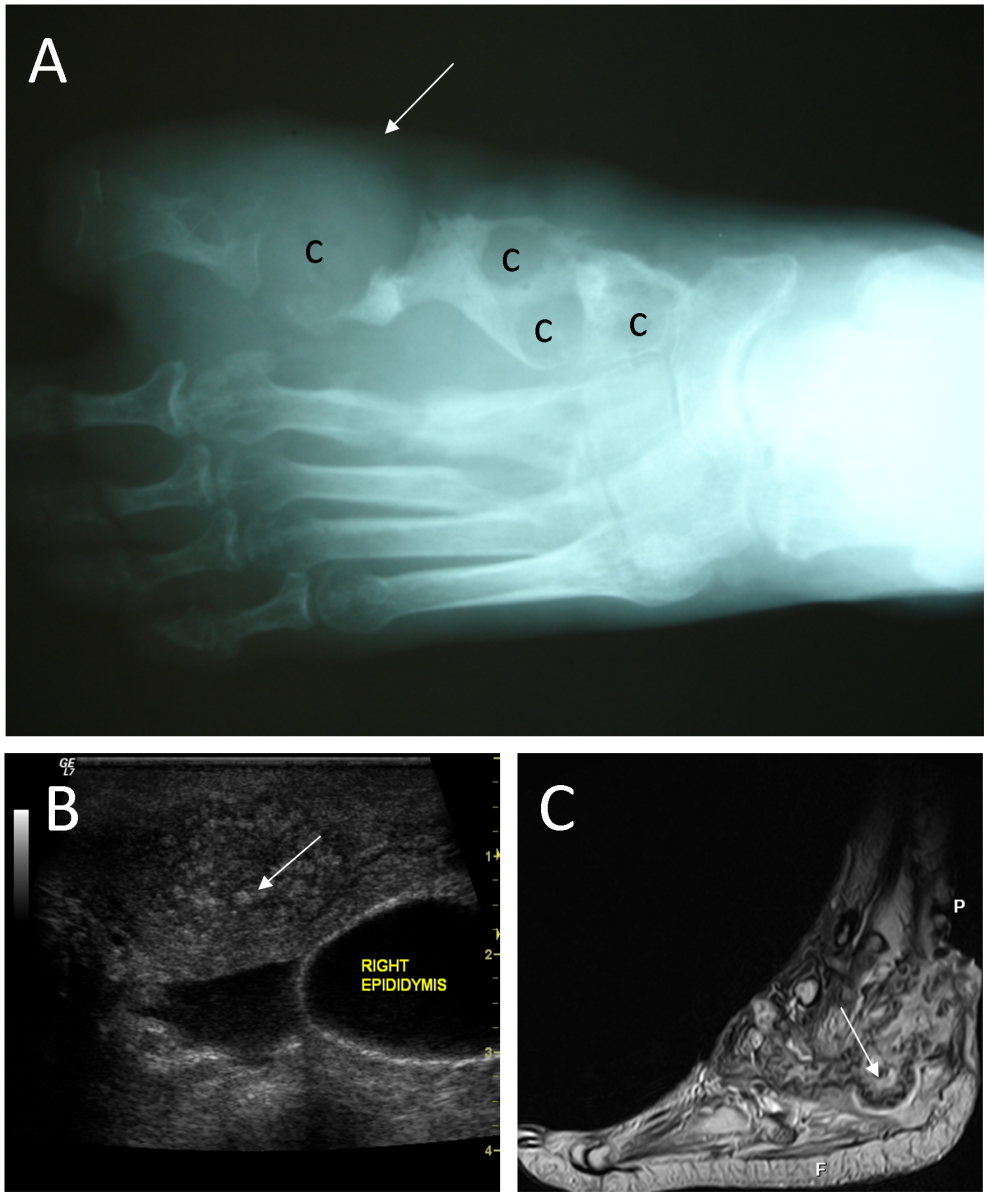
Imaging techniques used in mycetoma. A: A radiograph of the foot, showing soft tissue shadow (arrow), and multiple large cavities (c) in line with eumycetoma. B: A typical sonogram of scrotal eumycetoma. In this sonogram multiple cavities with thick walls and multiple hyper-reflective echoes (arrow) are seen, which are in line with grains. C: An MRI of the foot, showing massive soft tissue and bone destruction. In this MRI, grains appear as conglomerates of small (2–5 mm) round hyperintense lesions (arrow).

Conventional radiographs are used to identify the limits of lesions and to determine if bone is affected (Figure 2A). It is important to detect if bone is infected as nonsurgical cure is uncertain in such cases [6]. Several different radiological signs should be sought. Abd El Bagi suggested a radiographic classification of mycetoma to determine the extent of lesions (Table 1) based on radiographic records of 516 patients seen in the Mycetoma Research Centre, Khartoum, Sudan [7]. It is evident from this table that with classic radiology, multiple radiological changes can be detected. This method can be used to show the presence and extent of soft tissue granulomas [6] and to demonstrate bone invasion by the causative agent or the occurrence of pathological fractures [8]. Fractures, however, are uncommon; of the 517 patients seen in a diagnostic centre in Sudan, only 12 patients (2.3%) had pathological fractures [9]; no correlation was found between bone involvement and the duration of symptoms [6].

**Table 1:** Radiographic classification of bone involvement in mycetoma as described by Abd El Bagi [7].

Stage	Pattern of Spread	Effect	Findings
0	Limited to entry side	Soft-tissue swelling	No bone involvement
I	Expanding granuloma	Extrinsic pressure	Displacement or scalloping
II	Impending bone invasion	Bone irritation	Periosteal reaction or reactive sclerosis
III	Localized bone invasion	Erosion or cavitation	Solitary bone involvement
IV	Longitudinal spread	Joint involvement	Localized along single ray
V	Horizontal spread	Invasion of adjacent structures	Localized to forefoot, midfoot, or hindfoot
VI	Multidirectional spread	Total disruption	Multiple rays and multiple rows involved

Table adapted from Abd El Bagi et al. [7].

A more specific imaging technique used to detect mycetoma is ultrasonic imaging (Figure 2B) [10]. This technique is routinely used in the diagnosis of mycetoma. Mycetoma grains, capsules, and the accompanying inflammatory granulomata have a characteristic ultrasonic appearance [10]. Hyper-reflective echoes were noted only in mycetoma lesions and were recorded as fine (22%) or sharp (35%), numerous (50%) or few (17%), isolated (30%) or aggregated (17%), and diffuse (10%) or settled at the bottom of cavities (22%) [10]. However, when active sinuses are present in lesions, the identification of these hyper-reflective echoes are less clear [10]. Due to the presence of these hyper-reflective echoes, ultrasound imaging can be used to differentiate between mycetoma and non-mycetoma lesions and between actinomycetoma and eumycetoma [8], [10]. In eumycetoma lesions, the grains produce numerous sharp bright hyper-reflective echoes, which correspond to the black grains [8]. It is thought that the grain cement material is the origin of these sharp echoes. In actinomycetoma, the echoes obtained from grains are less distinct, probably due to their smaller size and consistency [8]. Although it is possible to distinguish between actinomycetoma and eumycetoma, it is difficult, if not impossible, to distinguish between different causative agents [8].

MRI is useful for visualizing soft tissue involvement and bone destruction (Figure 2C) [8], [11], [12]. With this technique, grains appear as conglomerates of small (2–5 mm) round hyperintense lesions, representing granulation tissue, surrounded by a low-signal-intensity rim [12]. By correlating MRI data with histological findings, Sarris et al. showed that the high-signal-intensity foci seen with MRI represented inflammatory granulomata, while the central low-signal-intensity dots were caused by grains [13]. The low-signal-intensity signs were named “dot-in-circle” and were seen in 80% of patients, which made this appearance highly indicative of mycetoma [12], [14]. In 2012, El Shamy and colleagues reported a new grading system for MRI diagnosis of mycetoma [15] in which a score was given for skin, subcutaneous tissue, muscle, and bone involvement (Table 2) [15]. Mycetoma lesions can be classified as mild (score 1–3), moderate (score 4–7), or severe (score 8–10) based on these scores. MRI was not suitable to discriminate actinomycetoma from eumycetoma, although actinomycetoma more often showed soft tissue microabscesses, bony periosteal reaction and reactive sclerosis, while eumycetoma frequently exhibited soft tissue macroabscesses with bone cavitation, such differences were not statistically significant [15].

**Table 2:** MRI classification of mycetoma lesions based on the Mycetoma Skin, Muscle, and Bone Grading (MSMB) system according to El Shamy et al. [15].

Score	MRI finding
*Skin and subcutaneous tissue*	
0	No skin or subcutaneous involvement
1	Obliteration of skin and fascial planes
2	Abscess formation
3	Formation of sinus tract without grains
4	Formation of sinus tract with grains
*Muscle*	
0	No muscle involvement
1	Muscle oedema
2	Formation of micro-abscess
3	Formation of macro-abscess
*Bone*	
0	No bone involvement
1	Bone oedema
2	Bone cavitation
3	Bone destruction

Table adapted from El Shamy et al. [15].

The merits of these imaging techniques are that they are fast, non-invasive, and can easily be used to detect the extent of lesions and help in planning appropriate treatment strategies. Of the various techniques discussed above, radiography and ultrasound imaging are the most applicable in low-income countries and can be used in local settings, provided that proper training is given. Radiography should normally be available in most peripheral hospitals, but not MRI, a situation that hinders the implementation of this latter method in standard diagnosis of mycetoma. When the technique is available, expertise is needed to differentiate between mycetoma and bone tuberculosis, granulomas and chronic bacterial osteomyelitis and soft tissue tumours [8].

## Cytology and Histology

**Figure 3:**
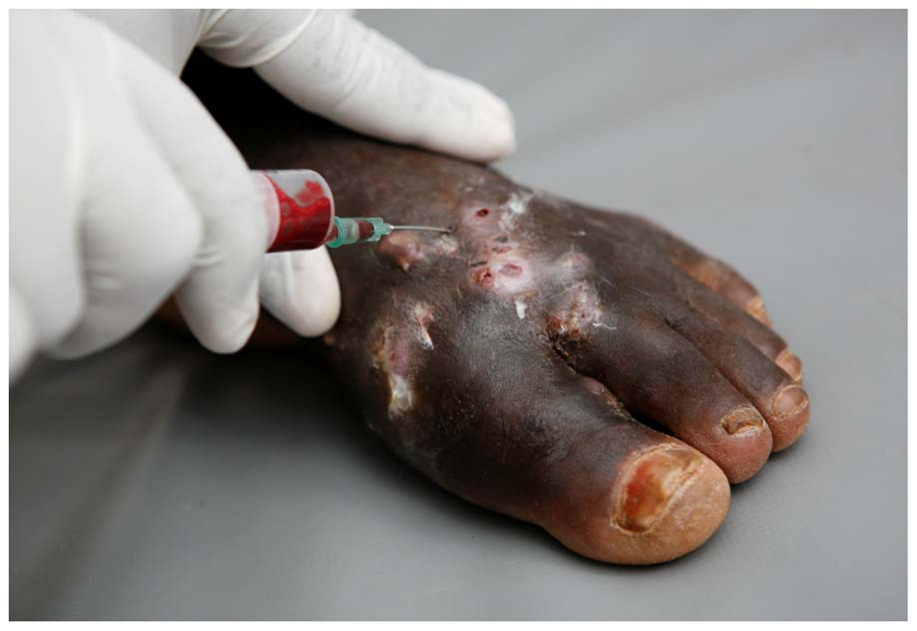
Obtaining grains via Fine Needle Aspiration.

Grains need to be isolated from lesions in order to identify causative agents of mycetoma to the species level. This is usually performed by a deep-seated biopsy or with fine needle aspiration cytology (FNAC) (Figure 3). The latter was described in 1991 and fully evaluated by Yousif and colleagues in 2010 [16]. In this technique, a fine needle attached to a syringe is inserted into the lesion by applying negative pressure and moving the needle up and down in at least three different directions so that sufficient aspirated material can be obtained. This material is usually bloody and is therefore left to clot, then examined either by eye or microscopically after fixation in 10% formalin-saline and staining with haematoxylin and eosin (H&E) [16]. Yousif examined 230 mycetoma patients using FNAC and the cell block technique to examine the grains of *Actinomadura madurae*, *Actinomadura pelletierii*, *Streptomyces somaliensis*, and *Madurella mycetomatis* and the tissue response to mycetoma. The technique had sensitivities of 87.5% and 85.7% for eumycetoma and actinomycetoma, respectively [16].

**Figure 4:**
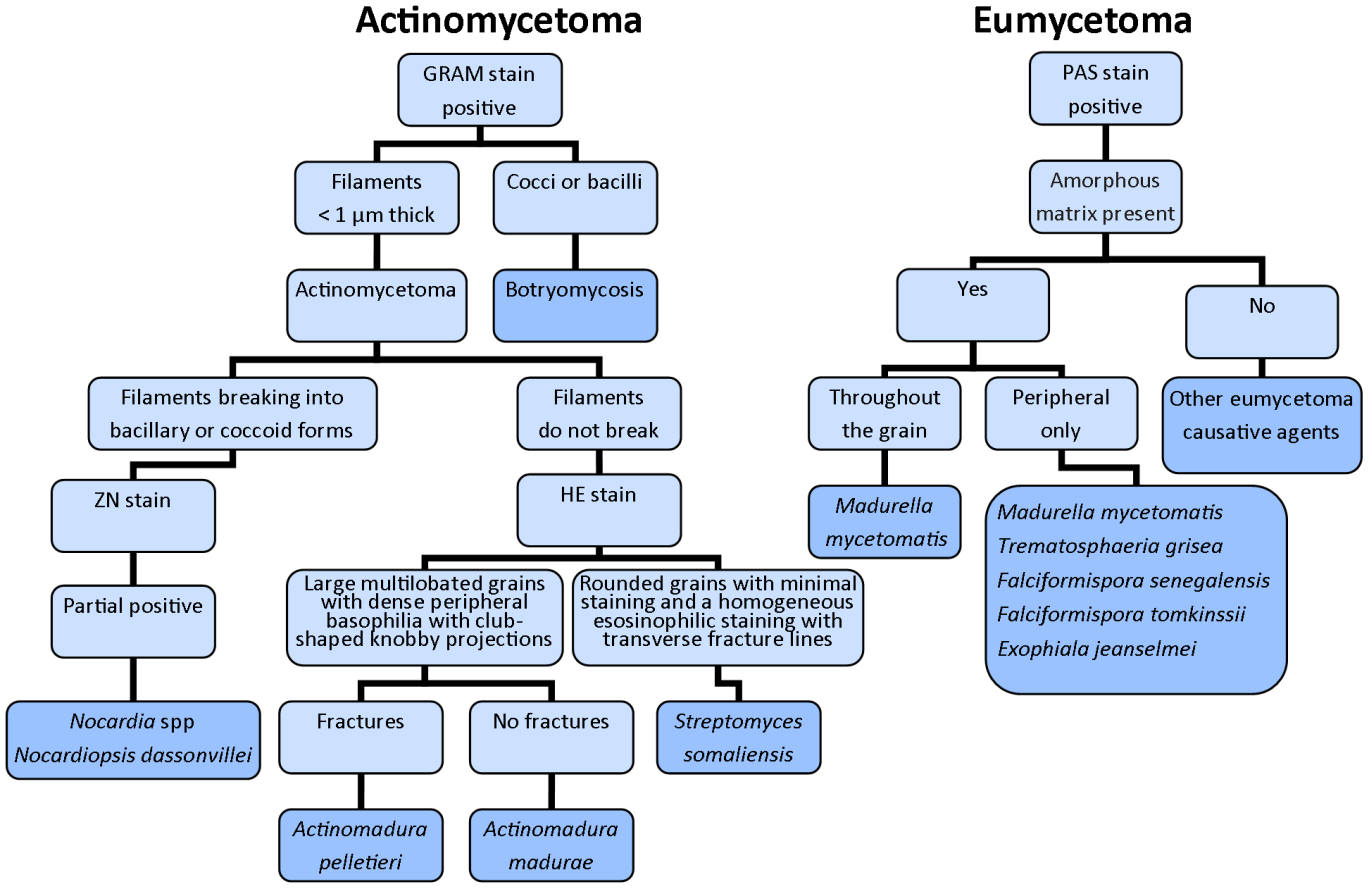
Flow diagram of histological identification of causative agents of mycetoma, based on references [18], [19], [23].

The identification of causative agents of mycetoma based on histology has been performed since the 1950s [17]. Grains can be identified in H&E stained sections (Figure 1), but special stains are essential for in-depth identification of the causative organisms [17], [18]. Gram stains, such as Gram Weigert, can be used to examine actinomycetoma tissue [18]. Periodic acid-Schiff (PAS), Gomori methenamine silver, and Gridley are the most useful stains for detecting hyphae and chlamydospores in eumycetoma grains [18]. Chufal and colleagues published a strategy in 2012 to identify causative agents based on histology (Figure 4) [19]. It can be seen from this figure that it is not possible to differentiate between *Nocardia* strains at the species level based on histology [20], [21]. Round, oval, or vermiform structures, which stain eosinophilic with haematoxylin, are found [17]. The delicate actinomycete filaments that may fragment into coccoid forms can be shown by Gram stain [17]. *A. pelletieri* grains, which stain red or dark blue with hematoxylin, consist of several lobules of geometric appearance with denticulate edges that resemble a broken dish [17]. In contrast, *A. madurae* grains have a central eosinophilic area surrounded by a densely packed zone of dark hematoxylin-stained filaments [17]. Peripherally, an eosin stained fringe with pseudoclub shaped structures can be observed [17].

Eumycetoma grains can be divided into black and pale grains. The most common causative agent is *M. mycetomatis* [4]. In histology sections, two types of grains of this agent are seen: filamentous and vesicular [22]. The filamentous type is the most common and consists of brown septate and branched hyphae that may be slightly more swollen towards the periphery [22]. The vesicular type is composed of unusually large cells which look like vesicles. Both types of grain can be found in the same lesion [22]. A brown granular cement material is present inside most of the grains [17]. In the black grains of *Falciformispora senegalensis* (synonym: *Leptoshaeria senegalensis*) and *Trematosphaeria grisea* (synonym: *Madurella grisea*) the centre is non-pigmented and cement is absent, whereas at the peripheries the grains are dark coloured and brown cement is present [17]. It is difficult to distinguish between *F. senegalensis* and *T. grisea* based on histology alone, but according to Mariat and Verghese, the borders of *F. senegalensis* grains are less even and more crenelated, the peripheral zones show larger vesicles while the hyphae at the periphery show a more irregular arrangement [17]. Pale grain eumycetoma caused by *Scedosporium boydii* (synonym: *Pseudallescheria boydii*), *Acremonium* spp. and *Fusarium* spp. are similar histopathologically and almost impossible to distinguish from each other [21]. There are some features that might be helpful in recognizing individual organisms [23]; for instance, in *Sc. boydii* vesicles are usually noted, though this feature cannot be used diagnostically [21], [23] as some *Acremonium kiliense* grains also have vesicles. In order to identify *Sc. boydii* more reliably on histological slides, Hagari and associates performed a nested PCR with *Sc. boydii* specific primers on paraffin-embedded histology slides (Table 3) [24].

**Table 3:** Primers used for molecular diagnostics.

Species	Target	Forward primer Sequence (5′→3′)	Reverse primer Sequence (5′→3′)	Probe Sequence (5′→3′)	Size (bp)	Reference
*Streptomyces* spp.	16S rRNA	AGAGTTTGATMTGGCTCAG	AAGGAGGTGWTCCARCC		1363 to 1521	[28]
*Nocardia* spp.	16S rRNA	ACCGACCACAAGGGG	GGTTGTAACCTCTTCGA		596	[54]
*Nocardia* spp.	16S rRNA	GCTTAACACATGCAAGTCG	GAATTCCAGTCTCCCCTG		606	[31]
*Nocardia* spp.	16S rRNA	AGAGTTTGATCCTGGCTCAG	ATTACCGCGGCTGCTGG		498	[59]
*Nocardia* spp.	16S-23S rRNA intergenic spacer	GTGCGGCTGGATCACCTCCT	GACAGCTCCCCGAGGCTTATCGCA		various	[58]
*Nocardia* spp.	*hsp65*	ACCAACGATGGTGTGTCCAT	CTTGTCGAACCGCATACCCT		441	[55], [59]
*Nocardia* spp.	*hsp65*	GTTGTCCTGGAGAAGAAGTGG	CTTGTCGAACCGCATACCCT		476	[59]
*Nocardia* spp.	*gyrB*	CTTCGCCAACACCATCAACAC	TGATGATCGACTGGACCTCG		610	[59]
*Nocardia* spp.	*gyrB*	CGAGGAGGGCTTCCGCGCGG	ATCGACTGGACCTCGTTGTTC		521	[59]
*Nocardia* spp.	*secA1*	GCGACGCCGAGTGGATGG	TTGGCCTTGATGGCGTTGTTC		483	[59]
*Nocardia* spp.	*secA1*	GCGACGCCGAGTGGATGG	GCGGACGATGTAGTCCTTGTC		520	[59]
*Nocardia* spp.	*rpoB*	CCGCTACAAGATCAACAAGAAGC	CCCGCGAGGACATCGTCG		737	[59]
*Nocardia* spp.	*rpoB*	CCGCTACAAGATCAACAAGAAGC	GGCGACGTACTCCATCTCCTC		780	[59]
*Nocardia* spp.	*rpoB*	CCCGCGAGGACATCGTCG	CCCGCGAGGACATCGTCG		655	[59]
*Nocardia* spp.	*rpoB*	CGAGTACCTGGTGCGYCTGC	TCGACCGGCGAGTTGGCCTG		599	[59]
Fungi	ITS	TTACGTCCCTGCCCTTTGTA	GCATTCCCAAACAACTCGACTC		Various	[64]
Fungi	ITS	TCCTCCGCTTATTGATATGC	GGAAGTAAAAGTCGTAACAAGG		Various	[67]
*M. mycetomatis*	ITS	AATGAGTTGGGCTTTAACGG	TCCCGGTAGTGTAGTGTCCCT		420	[67]
*Sc. boydii*	ITS	GAGGCAATAACAGGTCTGTGATGC	TTACTACGCAGAAGGCAA		800	[68]
*Sc. boydii*	ITS	AATCTTTGAACGCACATTG	TTACTACGCAGAAGGCAA		197	[24]
*Sc.boydii*	ITS	TGTCCGAGCGTCATTTC	TTACTACGCAGAAGGCAA		149	[24]
*Sc. boydii*	ITS	ATGGGCACCGAAGAAGCA	CGCGCAGACACGATA	*p* GGGTCGCGAAGACTCGCCGTA**gatca**TGCTTCTTCGGTGCCCAT**taccggtgcggatagctac**CGCGCAGACACGATA**gtcta**TTTCAGGGCCTACGGA	Various*	[70]
*Sc.boydii*	*BT2*	TGGCGAGCACGGTCTTG	ACATTCACGGCAGACACTGATT	FAM-TAGCAACGGAGTGTACGGAACCACCC-BBQ	96**	[69]

* Rolling Circle amplification assay, probe listed is the padlock probe to be used in this assay.

** quantitative real time PCR assay.

In summary, histology can be used to differentiate actinomycetoma and eumycetoma, while FNAC is a fast technique that may aid in choosing proper treatment. Histology cannot be relied upon for definitive species identification; hence, confirmation based on other techniques is needed.

## Culture and Phenotypic Characterisation of Colonies

Viable grains need to be taken from lesions in order to grow causative agents of mycetoma [25]. Since grains should be free of contaminants, a deep-seated surgical biopsy is needed [8], [25]. Grains collected from discharges of open sinuses are often non-viable and contaminated [8]. Some of the collected grains should be directly crushed in 10% KOH and used for microscopy; the remainder are used for culture [26].

Actinomycetoma grains can be identified using Gram and Ziehl-Neelsen (ZN) stains [8]. Gram staining can demonstrate the gram-positive hyphae of filamentous actinomycetes; the ZN stain is usually used for the identification of *Nocardia* spp. Fungal filaments can be stained with calcofluor white or cotton blue [27].

**Figure 5:**
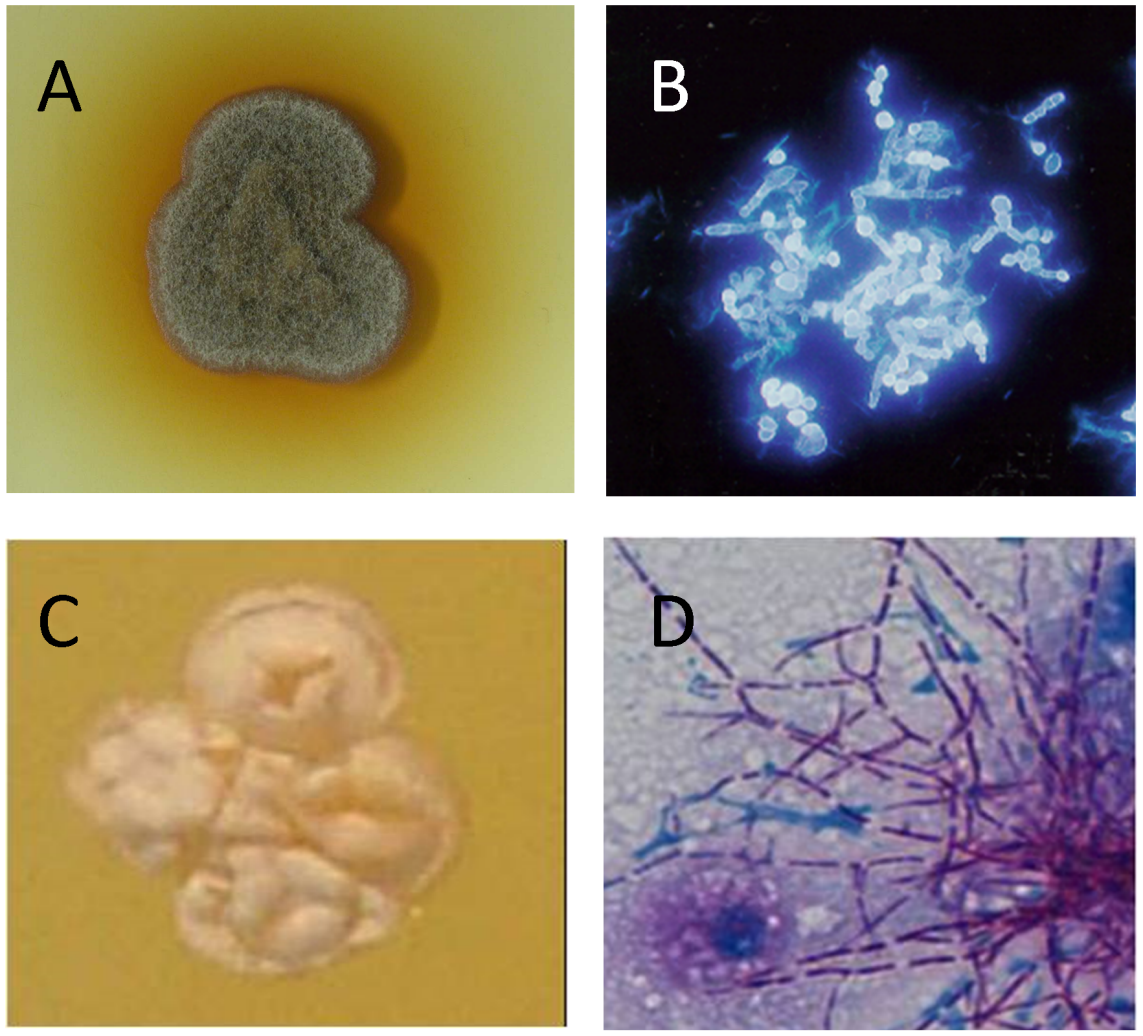
Identifying mycetoma causative agents by culture. A: *Madurella mycetomatis* grown on sabouraud agar. B: Microscopic appearance of *Madurella mycetomatis* stained with calcofluor white. C: *N. brasiliensis* colony. D: Microscopic appearance of *N. brasiliensis*.

In order to culture the causative agents, grains are usually washed several times in sterile saline, crushed with a sterile glass rod, and plated onto appropriate media [8], [25]. If direct examination of the grain reveals evidence of actinomycetes, isolation media should not contain antibiotics [18]. Blood, brain-heart infusion, Löwenstein, and modified Sabouraud agar supplemented with 0.5% yeast extract are commonly recommended for the isolation of actinomycetes [8], [18]. If direct examination of grains is indicative of eumycetoma, grains can be additionally washed with antibiotics and spread over Sabouraud agar plates supplemented with antibiotics. Commonly used antibiotics are gentamicin sulphate (400 µg/ml), penicillin G (20 U/ml), streptomycin (40 µg/ml), or chloramphenicol (50 µg/ml) [18]. It is also advisable to include a plate without antibiotics, since the growth of some *Madurella* and actinomycete strains appear to be inhibited by these antibiotics. Plates should be incubated at 25°C and 37°C irrespective of whether the causative agent is bacterial or fungal in origin (Figure 5).

After culture, presumptive species identification can be based on micromorphological and cultural properties [25]. Nocardiae are partially acid-alcohol fast positive and typically produce substrate and aerial hyphae that fragment into rod- and coccoid-like elements streptomycetes form a yellowish substrate mycelium and lack aerial hyphae, and *A. madurae* and *A. pelletieri* strains produce cream- and red-pigmented substrate mycelial respectively and on initial isolation tend to lack aerial hyphae [28], [29]. For eumycetoma, identification is mainly based on the morphology of the fruiting bodies (if present) [18]. For species such as *Madurella*, in which sporulation is usually not observed, the chance for misidentification is common. Furthermore, for *M. mycetomatis*, various colony morphologies have been observed, which makes identification even more troublesome [18]. Differentiation between *T. grisea* and *Medicopsis romeroi (synonym: Pyrenochaeta romeroi)* is also difficult. Although *T. grisea* is usually sterile, some isolates are known to produce abortive pycnidia like those formed by *Med. romeroi* [18]. Also differentiation of the various dematiaceous fungi, such as *Exopiala* spp., based solely on morphology is sometimes difficult, especially since some of these fungi only express yeast morphology on isolation, and initial subculture and conidiogenesis can only be induced after considerable manipulation [30].

For species identification of actinomycetes, phenotypic properties and assimilation tests can be performed, as exemplified by the use of API 20C, API ZYM and API Coryne kits for the identification of *Nocardia* species [31]–[33]. However, API 20C assimilation results have been found to be variable within *Nocardia* species, which makes definitive identification difficult [32]. When the assimilation of adonitol, galactose, glucose, glycerol, inositol, *N-acetyl*-D-glucosamine, and trehalose were considered reasonable, discrimination between *Nocardia* species was obtained, though results were not 100% specific [32]. In contrast, with the API ZYM system distinct enzymatic profiles were obtained for *Nocardia asteroides*, *Nocardia brasiliensis* and *Nocardia otitidiscaviarum*, though other pathogenic species of *Nocardia* could not be separated by this assay [33]. Further, *N. brasiliensis* was differentiated from *Nocardia farcinica* using API Coryne kits [31]. Antibiotic susceptibility patterns have also been used to identify various *Nocardia* spp., notably those based on amikacin, gentamicin, and tobramycin [32]. Since neither assimilation patterns nor susceptibility profiles alone were able to identify *Nocardia* species, Kiska and colleagues proposed a stepwise approach for species identification starting with susceptibility profiles and adding assimilation patterns and pigment production to the identification algorithm [32]. In this way, eight clinically relevant *Nocardia* species were identified [32]. However, the addition of several pathogenic species to the genus *Nocardia* makes it difficult to know whether such phenotypic tests can be weighted for identification in the future.

For the delineation of pathogenic *Streptomyces* spp. the following phenotypic properties are of value: production of β-glucuronidase; aesculin hydrolysis; degradation of adenine, casein and hypoxanthine; growth on adonitol, glycerol, glycogen, *meso*-inositol, D-raffinose, L-rhamnose, D-turanose, D-xylose and L-aspartic acid as sole carbon sources [28]. Furthermore, growth at 50°C and pH 11.0 provide additional data of diagnostic value [28]. *A. madurae* and *A. pelletieri* can be separated from most other actinomycetes by the detection of chymotrypsin using the API ZYM system. In addition, *A. madurae* was found to be positive for α-glucosidase and negative for *N*-acetyl-β-glucosaminidase, while for *A. pelletieri* the opposite was found [33].

Limited information is available on phenotypic properties and assimilation patterns for eumycetoma agents; therefore, these pathogens are still mainly identified solely by morphology. *T. grisea* can be distinguished from *M. mycetomatis* based on the presence of a secreted pigment and the assimilation of lactose, but not sucrose by *M. mycetomatis* [27]. It remains to be seen whether this simple system can be used to identify recently classified *Madurella* species, namely *M. fahalii*, *M. pseudomycetomatis* and *M. tropicana* using API 20C AUX kits [34]. *M. pseudomycetomatis* was found to assimilate arabinose, cellulose, glucose, maltose, trehalose, and xylose, though such tests have yet to be applied to the two other *Madurella* species [34].

Similarly, for *Sc. boydii*, an array of physiology and assimilation patterns have been determined [35] but have not been routinely used to distinguish other species. Carbohydrate and nitrate assimilation have been determined for *Exophiala* spp., though it is very difficult to differentiate between these species solely on this basis [30]. *E. dermatitidis* and *E. jeanselmei* have very similar assimilation patterns and can only be differentiated from one another based on NaNO_3_ assimilation [30].

In summary, culture methods are still considered to be the gold standard in species identification of the causal agents of mycetoma; however, some agents are difficult to identify by morphology alone. Furthermore, most culture methods are time consuming, contamination is common, and experience is needed to read results accurately.

## Skin Tests

In order to develop a fast diagnostic tool, skin tests were developed in the past and applied with some success. When protein derivatives of *N. brasiliensis* were intradermally administered in a dose of 0.2 µg in 0.1 ml, all of the patients infected with this organism reacted positively to this antigen with indurations of >6 mm in diameter, while none of the tuberculosis patients or healthy adults showed a positive reaction [36]. However, a single mycetoma patient infected with *A. madurae* did not give a positive reaction in the skin test. When culture filtrate antigens were prepared in 0.1 ml quantities from *A. pelletieri*, *S. somaliensis* and *M. mycetomatis*, positive skin reactions were found in patients infected with either *A. madurae* or *S. somaliensis* using the *A. pelletieri* and *S. somaliensis* antigens after 5 hours and after 24 hours, but not against *M. mycetomatis* [37]. Four out of the 13 *M. mycetomatis* eumycetoma patients gave a positive skin reaction against the *M. mycetomatis* antigen within 5 hours, though only one gave a positive reaction after 24 hours [37]. Furthermore, five patients reacted with the *A. pelletieri and S. somaliensis* antigens after 5 hours but not after 24 hours, demonstrating that this skin test was not useful in diagnosing eumycetoma [37].

## Serology

Due to the invasive procedures needed to obtain grains and the long incubation times needed for identification of causative agents, attempts have been made to develop serological assays for the diagnosis of mycetoma.

Immunodiffusion (ID), counter-immuno-electrophoresis (CIE), and Enzyme Linked ImmunoSorbent Assays (ELISA) have been developed to detect mycetoma caused by *A. madurae*, *A. pelletieri*, and *S. somaliensis* using crude cytoplasmic antigens of these agents [38]–[41]. CIE and immunodiffusion are two techniques that are based on the formation of antibody-antigen precipitin lines in agar gels [38], [42]. Gumaa and Mahgoub demonstrated that from 200 sera negative by immunodiffusion, 76 sera reacted in the CIE; hence, they concluded that CIE was more sensitive than ID in the diagnosis of mycetoma [38]. McLaren compared ELISA with CIE and found that while the former was more sensitive, it gave weak positive reactions in some of the Egyptian and Sudanese controls. This background reaction was reduced when the cytoplasmatic antigens were centrifuged for 30 minutes at 20,000 g, indicating that the pure antigens are necessary to produce a reliable diagnostic serological tool [41].

Serological tests have been developed for *N. asteroides* and *N. brasiliensis* using purified antigens from culture supernatants of these organisms. SDS-PAGE was used to demonstrate that a 54–55 kDA antigen found in culture media was present in *N. asteroides*, *N. brasiliensis*, and *N. otitidiscaviarum* [43]. From dot blot and western blot analysis it was demonstrated that the antigen reacted with sera from *N. brasiliensis-*, *N. asteroides-*, and *R. rhodochrous*-infected patients, but not with sera from healthy controls [43]. Surprisingly, in the dot blot analysis one out of two *A. madurae*-positive patients also reacted with this antigen [43]. In the Western blot analysis, sera from nocardiosis patients and one out of five tuberculosis patients also reacted positively.

More discriminatory antigens were identified by Vera-Cabrera and Salinas-Carmona and colleagues [43], [44]. They identified immunodominant proteins of 24 kDa (named P24) and 61 kDa (named P61) by SDS-PAGE and purified them by Sephadex purification and dialysis, respectively. In 1993, an ELISA was developed with the purified p24 protein and tested against mycetoma, leprosy, and tuberculosis patients. All 26 mycetoma patients gave absorbance values equal to or above 0.3, whereas the serum samples from 53 tuberculosis and leprosy patients or from healthy controls had absorbance values below this level. Several sera were available for analysis of five mycetoma patients during, before, and after therapy. During active disease, absorbance values were always above 0.3, but when patients were treated and classified as cured, the absorbance levels dropped below this level. This showed that the assay could be useful for monitoring therapeutic responses [44]. Furthermore, antibody titres dropped in patients who were cured and increased in those who presented reactivation [44].

Serological assays have been developed to detect infections caused by *M. mycetomatis* and *Sc. boydii*. An indirect hemagglutination assay (IHA) and an ID assay with crude antigens were developed for *Sc. boydii* [39], [45]. In the IHA, a partially purified polysaccharide antigen isolated from *Sc. boydii* was coupled to sheep erythrocytes, and a serial two-fold dilution of the patient serum was added [45]. It appeared that five patients had antibody levels ranging from 1∶16 to 1∶512, and the healthy controls' antibody levels ranged from 1∶4 to 1∶32 in endemic areas.

The ID and CIE methods have been more widely used, especially for *M. mycetomatis* [38], [39]. In these assays, crude cytoplasmatic antigens extracted only with saline or precipitated by acetone are used [39]. Positive precipitation bands were found in all sera from patients with proven and presumed *M. mycetomatis* mycetoma when *M. mycetomatis* antigens were used and the same was true for *Sc. boydii* [39]. Later it appeared that the immunodiffusion assay could be negative in some patients with mycetoma, especially ones receiving treatment or with early lesions [38]. CIE appeared to be superior to ID in terms of sensitivity for eumycetoma [38], [39], as was the case for actinomycetoma.

ELISAs have been developed to define if there are more sensitive and specific tools to determine the antibody response to mycetoma antigens. The ELISA developed by Wethered and colleagues was produced with crude extracts and could discriminate between patients and controls [46]. The ELISA produced by McLaren and colleagues was less reliable: out of nine healthy controls tested, four were positive with no clinical sign of mycetoma [41]. Taha and colleagues demonstrated that with their ELISA, both mycetoma patients and uninfected controls living in endemic areas had antibodies formed against mycetoma agents [40]. Consequently, in endemic areas, only CIE has been used for the diagnosis of mycetoma. Recently, the Mycetoma Research Centre in Khartoum stopped using this assay because the lack of standardization of the antigen preparation resulted in a variation in the outcome of this diagnostic tool. The partially purified antigens for both CIE and other serological tools are prepared by grinding mycelia in saline, disintegrating them ultrasonically, and collecting the cytoplasmatic proteins by centrifugation [26], [46], [47]. Production of such antigens may result in variation between tests. A serological assay based on a recombinant antigen is to be preferred.

Recombinant antigens have been produced for *M. mycetomatis*. The only serological tools which use recombinant antigens are the ELISA described by van de Sande and colleagues [48] and the Luminex described by de Klerk and associates [49]. The recombinant translationally controlled tumour protein (TCTP) was included in both assays. The Luminex assay also included recombinant fructose-bisphosphate aldolase (FBA) and pyruvate kinase (PK) antigens [48], [49]. With these recombinant antigens it was noted that although patients gave higher antibody levels, healthy controls also produce antibodies against them, making it impractical to use these recombinant antigens as diagnostic tools.

In summary, serological tools provide a cheap and relatively fast way to identify mycetoma causative agents, though to achieve reliable results it is necessary to use standardized antigen preparations. For *N. brasiliensis* a reliable serological tool has been developed with the purified p24 protein, but for the other causative agents of mycetoma reliable serological diagnostic tools are not yet available.

## Molecular Techniques

Difficulties associated with the accurate identification of the known causative agents of actinomycetoma using phenotypic criteria promoted the development and application of molecular diagnostic procedures. Reliable identification of causal agents to the genera *Actinomadura*, *Nocardia*, and *Streptomyces* can be achieved by PCR and sequencing of conserved genes, notably, by comparison of almost complete 16S rRNA gene sequences of isolates against corresponding sequences of their phylogenetic neighbours drawn from databases using software such as EzTaxon-e [50] and appropriate clustering algorithms. In contrast, identification of pathogenic actinomadurae, nocardiae, and streptomycetes to the rank of species remains problematic.

Several molecular methods have been developed for the identification of aerobic, filamentous, clinically significant actinomycetes, notably nocardiae. These include the use of PCR coupled with restriction endonuclease analysis of the resultant products [51]–[55] and PCR of randomly amplified polymorphic DNA fingerprints [56]. Similar approaches based on 16S rRNA gene polymorphisms have also been employed [57].

Wehrhahn and colleagues developed a PCR procedure for identification and subtyping of *Nocardia* species based on the 16S–23S intergenic spacer region that resulted in the generation of amplicons of different sizes; these were analysed using a cost-effective sequencer-based capillary gel electrophoretic system [58]. This method was used to correctly identify 142 out of the 145 tested isolates to the correct species [58]. McTaggart developed a multilocus sequence analysis (MLSA) based on sequencing of 16S rRNA genes, the β subunit of type II DNA topoisomerase (*gyrB*) genes, the subunit A of SecA preprotein translocase (*secA1*) genes, *hsp65* genes, and RNA polymerase (*rpoB*) genes [59]. This method was used to reliably identify *Nocardia* species, but the three-loci (*gyrB-*16S-*secA1*) or four-loci (*gyrB-16S-sefcA1-hsp65*) MLSA was nearly as reliable and is more amenable for use in the diagnostic clinical microbiology laboratories [59].

Molecular diagnostic procedures have led to significant improvements in the identification of aerobic, pathogenic actinomycetes, though there is evidence that the results of such studies need to be interpreted with care if the misidentification of unusual isolates is to be avoided [60]–[62]. Again, the long-term diagnostic value of current molecular-based procedures is difficult to foresee, given the need to apply them to an ever-expanding number of *Actinomadura*, *Nocardia*, and *Streptomyces* species. This problem is compounded by evidence that key pathogenic species, such as *A. madurae* and *N. asteroides*, are heterogeneous [29], [63]. Consequently, unusual or potentially novel causal agents of actinomycetoma need to be compared with their immediate neighbour(s) in 16S rRNA gene trees prior to the application of additional diagnostic procedures known to have traction at the rank of species.

The molecular identification of the causal agents of eumycetoma is mainly based on the internal transcribed spacer (ITS) that is located between the 18S and 28S genes. By using the pan-fungal ITS primers and by either sequencing or digesting the resulting PCR product with restriction-endonucleases, causative agents can be identified to the species level [64]–[67]. A PCR developed for *M. mycetomatis* using primers located within the ITS region appears to be specific, as amplification was not detected when DNA from *T. grisea*, *Biatriospora mackinnonii* (synonym: *Pyrenochaeta mackinnonii*), and *Med. romeroi* were used [67]. Molecular diagnostic tools have been developed for *Sc. boydii*, but most of these assays are based on the β-tubulin gene (*BT2*) and not on the ITS gene, as the *BT2* gene is more discriminatory. The PCR developed for the ITS region by Wedde and associates, for instance, not only amplified *Sc. boydii* but also *Pseudallescheria augusta* and *Pseudallescheria ellipsoidea* [68]. A quantitative real time PCR (qPCR) procedure with a probe specific for *Sc. boydii* and *Scedosporium apiospermum* (Table 3) has been developed, though some cross-reactivity was observed with *Scedosporium minutispora* [69].

For endemic countries, with only limited resources, species identification based on PCR and sequencing or PCR and restriction endonuclease analyses is still expensive; hence, efforts have been made to perform species identification of the causal agents of mycetoma through cheaper methods. One such method is the Rolling Circle Amplification (RCA) technique, which is designed to identify *Sc. boydii* and is based on the amplification of the ITS region with pan-fungal primers (Table 3). After amplification, species identification is performed by hybridizing species-specific padlock probes to the amplified ITS product and ligated [70]. By adding the pan-fungal RCA primers and the *Bst* DNA polymerase, species-specific amplification is performed for 60 minutes at 65°C. If amplification occurs, the result can be analysed on gels. Unfortunately this procedure still relies on the amplification of the ITS gene, and for some endemic countries, the thermocyclers needed to perform the PCR are not routinely available. The Loop-mediated Isothermal Amplification (LAMP) method was developed for the identification of *Sc. boydii* (Table 4) [69]. Genomic DNA can be amplified using this technique by adding the six primers listed in Table 4 and *Bst* DNA polymerase and incubating for 60 minutes at an isothermal temperature of 63°C [69]. The resulting product can be visualised either by Gel-electrophoresis or by visual observation after the addition of SYBR green. This method is suitable for species identification in endemic countries, especially since only simple extraction methods are needed, based on Whatman FTA [69].

**Table 4:** Primers used for LAMP.

Species	Target	Primer Sequence (5′→3′)	Reference
*Scedosporium boydii*	*BT2*	ATGGCACTTCTGAACTCCAG	[69]
		CGAGATCTACAAGGACAGCG	
		CTTGAGCGCATGAGCGTT	
		AACCAGCCCGTGGTTTGAACC	
		TTTGTTGCCCGAAGCCTAT	
		GTGTGGTGTCATCCAGCCTCC	

## Conclusions

Diagnostic tools have been developed to aid in the diagnosis of mycetoma, but most of them cannot be used alone. Clinical examination and different imaging techniques can determine the extent of the disease and, to some extent, its type. A first indication of the causative agent can be obtained by isolating grains from lesions with a fine needle aspirate. Further assessment can be achieved by simply looking at the color of grains. More reliable data can be gleaned by looking at grains histologically, but the definite identification of causative agents of mycetoma should be done only after culture and preferably by using appropriate molecular tools.

The main drawback of molecular-based diagnostic tools is that they usually have to be performed in large diagnostic centers, such as referral hospitals in Mexico or specialized clinics in Sudan. Since most of the patients do not live near such health facilities, expensive travel is often necessary. Efforts need to be made to develop easy bedside and field-based diagnostic tools to aid in the identification of causative agents to the species level and to prescribe proper treatment accordingly. For this, comparative genomics of the already sequenced genomes of *N. brasiliensis* [71] and *S. somaliensis* [72], and the genomes of other causative agents which might be sequenced in the future will provide essential data for developing species-specific identification tools. Procedures already useful for such bedside implications include the LAMP-based molecular diagnostic tools for *Sc. boydii* and agglutination based serological tools.

**Key Learning Points**

- Since both actinomycetes and fungi are implicated as causative agents, it is important to distinguish them in order to ensure that correct treatments are given.
- An initial diagnosis is made after clinical assessment. Clinical examination alone does not identify the causative organism, nor does it detect the spread of disease along the different tissue planes and bone. Imaging techniques can be used to detect the extent of lesions and help in planning appropriate treatment strategies.
- Culture methods are still considered to be the gold standard in species identification of the causal agents of mycetoma; however, some agents are difficult to identify by morphology alone. Furthermore, most culture methods are time consuming, contamination is common, and experience is needed to read results accurately.
- Histology, cytology, skin test, and serology have been developed to aid in species identification, but most of these assays appeared not specific enough to identify the causative agent to the species level.
- At the moment only molecular methods are able to identify the causative agent to the species level reliably. However these methods are usually too expensive for the endemic countries. Therefore fast and cheap diagnostic tools need to be developed to enable early case detection, thereby enhancing the change of a better therapeutic outcome.

**Top Five Papers in the Field**

- Abd El Bagi ME (2003) New radiographic classification of bone involvement in pedal mycetoma. AJR Am J Roentgenol 180: 665–668.
- Salinas-Carmona MC, Welsh O, Casillas SM (1993) Enzyme-linked immunosorbent assay for serological diagnosis of *Nocardia brasiliensis* and clinical correlation with mycetoma infections. J Clin Microbiol 31: 2901–2906.
- Gumaa SA, Mahgoub ES (1975) Counterimmunoelectrophoresis in the diagnosis of mycetoma and its sensitivity as compared to immunodiffusion. Sabouraudia 13: 309–315.
- Laurent FJ, Provost F, Boiron P (1999) Rapid identification of clinically relevant *Nocardia* species to genus level by 16S rRNA gene PCR. J Clin Microbiol 37: 99–102.
- van de Sande WWJ, Fahal AH, de Hoog GS, Van Belkum A (2011) *Madurella*. In: Liu D, editor. Molecular detection of human fungal pathogens. Boca Raton: CRC Press, Taylor & Francis Group. pp. 117–128.
